# Quantitative Mapping of Matrix Content and Distribution across the Ligament-to-Bone Insertion

**DOI:** 10.1371/journal.pone.0074349

**Published:** 2013-09-03

**Authors:** Jeffrey P. Spalazzi, Adele L. Boskey, Nancy Pleshko, Helen H. Lu

**Affiliations:** 1 Biomaterials and Interface Tissue Engineering Laboratory, Department of Biomedical Engineering, Columbia University, New York, New York, United States of America; 2 Musculoskeletal Integrity Program, Hospital for Special Surgery, New York, New York, United States of America; 3 College of Dental Medicine, Columbia University, New York, New York, United States of America; University of Pittsburgh, United States of America

## Abstract

The interface between bone and connective tissues such as the Anterior Cruciate Ligament (ACL) constitutes a complex transition traversing multiple tissue regions, including non-calcified and calcified fibrocartilage, which integrates and enables load transfer between otherwise structurally and functionally distinct tissue types. The objective of this study was to investigate region-dependent changes in collagen, proteoglycan and mineral distribution, as well as collagen orientation, across the ligament-to-bone insertion site using Fourier transform infrared spectroscopic imaging (FTIR-I). Insertion site-related differences in matrix content were also evaluated by comparing tibial and femoral entheses. Both region- and site-related changes were observed. Collagen content was higher in the ligament and bone regions, while decreasing across the fibrocartilage interface. Moreover, interfacial collagen fibrils were aligned parallel to the ligament-bone interface near the ligament region, assuming a more random orientation through the bulk of the interface. Proteoglycan content was uniform on average across the insertion, while its distribution was relatively less variable at the tibial compared to the femoral insertion. Mineral was only detected in the calcified interface region, and its content increased exponentially across the mineralized fibrocartilage region toward bone. In addition to new insights into matrix composition and organization across the complex multi-tissue junction, findings from this study provide critical benchmarks for the regeneration of soft tissue-to-bone interfaces and integrative soft tissue repair.

## Introduction

The interface between soft tissue and bone is essential for physiologic musculoskeletal motion, and serves to integrate and minimize stress concentrations between distinct tissue types. A functional interface with bone is especially important for the Anterior Cruciate Ligament (ACL), the primary tibiofemoral intra-articular ligament and joint stabilizer [1]. The biomechanical functionality of the ACL is rooted in the organized ligament structure, with biological fixation to bone facilitated by complex fibrocartilaginous insertions into the femur and tibia [1–3]. Specifically, the interface between ACL and bone is divided into four distinct yet continuous tissue regions, with region-specific distributions in cell type and matrix composition [3–10]. The first region is the ligament proper, in which fibroblasts reside in a matrix rich in types I and III collagen. Contiguous with the ligament is the fibrocartilage interface, which is subdivided into non-calcified and calcified regions. The non-mineralized fibrocartilage (NFC) is composed of fibrochondrocytes in a matrix consisting of types I and II collagen, while hypertrophic chondrocytes within a type X collagen-containing matrix are found in the mineralized fibrocartilage (MFC) region, which finally transitions into bone. This region-dependent matrix organization and the subdivision of the interface into non-calcified and calcified regions lead to a gradual increase in mechanical properties across the interface regions, thereby minimizing stress concentrations and allowing for effective load transfer from ligament to bone [3,11,12].

This complex ligament-to-bone transition, however, is not maintained or re-established following ACL reconstruction. Absence of this functional interface may compromise graft stability and long-term clinical outcome [13–16]. Developing an understanding of the structure-function relationship inherent to the ACL-bone interface is a prerequisite for its regeneration [13–17] and ultimately the seamless formation of complex tissues. While the ACL insertions have been extensively characterized using histological [3,6–8,10] and, more recently, mechanical [11,18] analyses, there is a critical need for quantitative assessment of the various components contributing to regional matrix organization. Therefore, the goal of this study is to utilize Fourier transform infrared spectroscopic imaging (FTIR-I) [19] to construct quantitative spectroscopic maps of matrix composition, distribution, and organization. An in-depth understanding of interface matrix distribution and organization is essential for relating structure to function, and for establishing benchmark criteria for interface tissue engineering.

Spectroscopic imaging methods such as FTIR-I [20–24] and Raman [25,26] spectroscopy have been used to analyze musculoskeletal tissues. This study utilizes FTIR-I as it is a sensitive and high-throughput imaging modality which is capable of mapping the matrix composition and distribution of a relatively large sample area, thereby making it well suited for examining multi-tissue regions. Published studies confirm that spectroscopic mapping correlates directly with histological analysis [21,27], albeit without the inherent shortcomings of histology (e.g. staining solution variations and subjective interpretation). Moreover, FTIR-I analyses have been shown to correlate with quantitative biochemical analysis of proteoglycans [21], collagen and aggrecan model compounds with varying proportions of the two constituents [28], and mineral ash weight [29]. Specifically, high correlation has been reported by Kim et al. between FTIR-I measurements and total proteoglycan content in tissue engineered cartilage [21]. In addition, FTIR-I analysis of canine bone revealed that the mineral-to-matrix peak area ratio correlates linearly with tissue ash weight [29]. Furthermore, the ratio of amide I to amide II band areas can be used to determine collagen orientation [24] as the transition moments for the amide I and amide II bond vibrations are approximately perpendicular [23]. Therefore, in addition to being a well-validated method, FTIR-I is advantageous in that the distribution and organization of key matrix components can be quantified concurrently on the same sample, making it an efficient and informative method for the characterization of multi-tissue transitions such as the ACL-to-bone interface.

The objective of this study is to characterize the complex matrix organization at the ACL insertions using FTIR-I, mapping region-dependent changes in collagen, proteoglycan, and mineral content as well as collagen orientation across the multi-tissue transition. As this is the first reported study to utilize FTIR-I for the characterization of the ACL-to-bone interface, image acquisition as a function of sample preparation (sagittal vs. transverse) will be compared. The second objective of this study is to identify any insertion site-dependent changes in matrix content and distribution by comparing spectroscopic mapping of the femoral and tibial insertions. Both region- and insertion site-dependent differences are anticipated across the interface regions. Findings from this study will lead to new insights into matrix composition and organization at this critical soft tissue-to-bone junction, while providing the much needed benchmarks for interface regeneration and integrative soft tissue repair.

## Methods

### Sample Isolation

The insertion site samples were isolated from neonatal bovine tibiofemoral joints obtained from a local abattoir (n = 6, Green Village Packing Company, Green Village, New Jersey, USA). After removal of surrounding muscle, subcutaneous fascia, and collateral ligaments, the patella and patellar tendon were removed, followed by the underlying adipose tissue as well as both medial and lateral menisci. The cruciate ligaments were transected, and both the femoral and tibial ACL insertions were identified and excised. Transverse or sagittal incisions approximately 7 mm apart were made through the insertion specimens to isolate samples containing regions of ligament, fibrocartilage, and bone.

### Sample Preparation for Matrix and Mineral Analysis

Both decalcified and non-decalcified samples were evaluated in this study. To prepare the decalcified samples, transverse and sagittal samples of the insertions (n=3 per insertion site from each of three specimens) were immediately fixed with 80% ethanol and 1% cetylpyridinium chloride (CPC, Sigma, St. Louis, Missouri, USA) for 24 hours following the methods of Bi et al. [24]. Ethanol was chosen as the fixative to minimize the effect of fixation on the IR spectral parameters [30], while CPC was added to preserve proteoglycans [27,31]. Following fixation, samples were rinsed in distilled water to remove the CPC, and subsequently demineralized in tris-hydroxymethylaminomethane (Tris, Sigma) buffer containing 10% ethylenediaminetetraacetic acid (EDTA, Sigma) for three weeks, after which the insertion samples were dehydrated using an ethanol series, cleared with xylenes, and embedded in paraffin (Fisher Scientific, Pittsburgh, Pennsylvania, USA). The embedded samples were sectioned using a microtome (Reichert-Jung RM 2030 Microtome, Leica, Bannockburn, Illinois, USA), and sections (7 µm) were placed immediately onto barium fluoride infrared-transmissive windows (Spectral Systems, Hopewell Junction, New York, USA). The sections were subsequently deparaffinized in xylenes to minimize paraffin interference with the IR spectra, rehydrated with an ethanol series, and then dried overnight under vacuum. A second barium fluoride window was placed over the sample prior to infrared analysis.

Non-decalcified samples were used for mineral analysis. To this end, transverse insertion samples (n=3 per insertion site from each of three specimens) were fixed after isolation with 90% ethanol for 24 hours [30,32]. Following fixation, samples were embedded in polymethylmethacrylate (PMMA) using a modification of methods described by Erben [33]. Ethanol fixation and PMMA embedding have been shown by Aparicio et al. to minimize interference that would otherwise be obtained in the acquired spectra from other fixation and embedding methods [30], and have been employed successfully in prior infrared evaluations of mineralized tissues [34–36]. The samples were then sectioned (2 μm) with a sliding microtome (SM2500S, Leica Microsystems Inc., Deerfield, Illinois, USA) fitted with a tungsten carbide blade (Delaware Diamond Knives Inc., Wilmington, Delaware, USA). Individual sections were dried and placed between barium fluoride windows for analysis.

### Fourier Transform Infrared Spectroscopic Imaging (FTIR-I)

**Figure 1:**
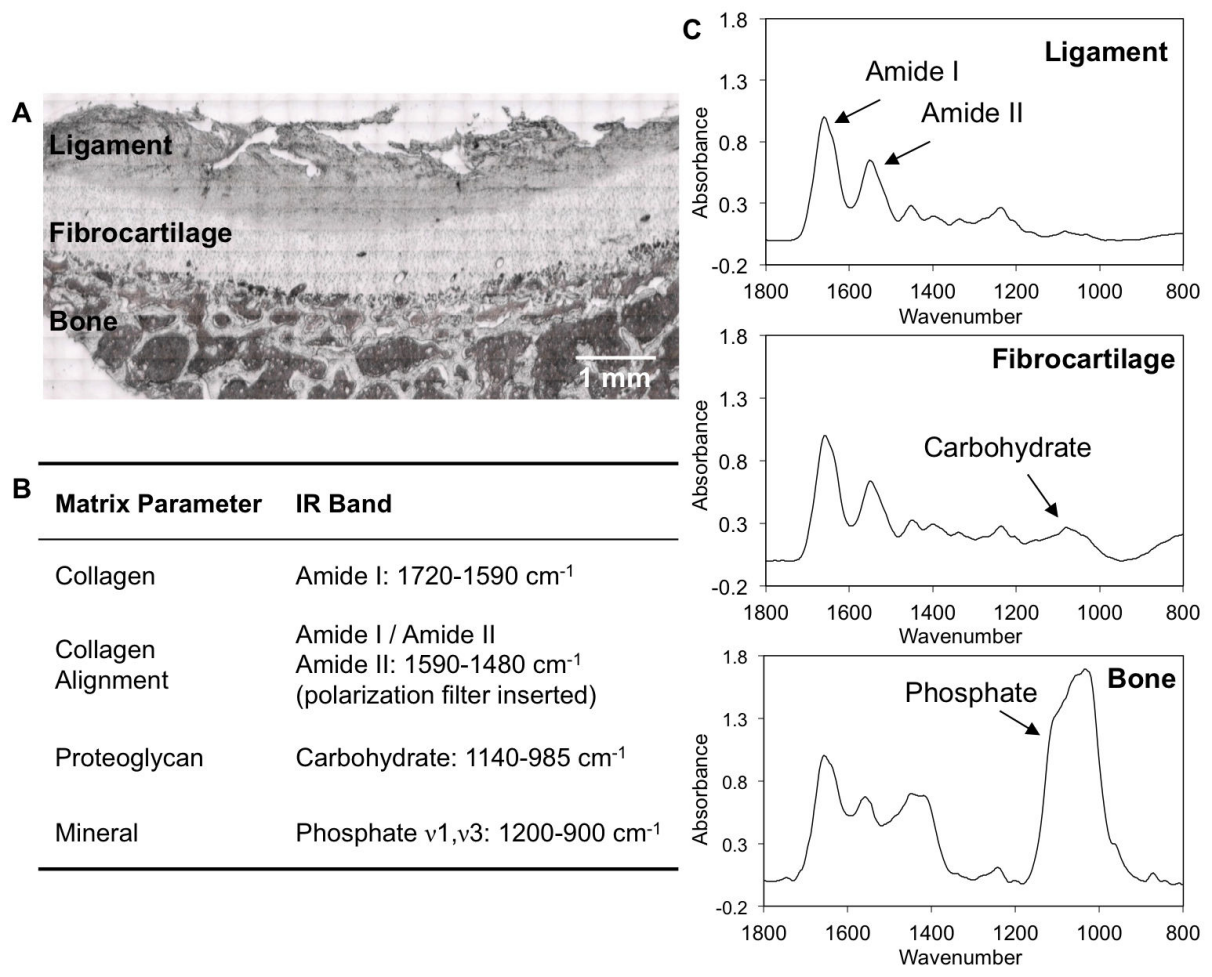
ACL insertion matrix parameters and analyzed IR bands. A) Light microscopy image of a decalcified ACL insertion from which the spectral scan areas were selected. B) IR peaks analysed include amide I, amide II, phosphate, and carbohydrate bands to quantify collagen content, collagen orientation, mineral distribution, and proteoglycan content, respectively. C) Representative infrared spectra of the tissue regions found across the ACL-to-bone insertion (ligament, fibrocartilage, and bone) normalized by the amide I peak.

Fourier transform infrared imaging (FTIR-I) analysis was performed using an FTIR spectrometer (Spectrum 100, PerkinElmer, Waltham, Massachusetts, USA) coupled to an FTIR microscope imaging system (Spotlight 300, PerkinElmer). Spectra were acquired between 2000-800 cm^-1^ with a spectral resolution of 8 cm^-1^ and a spatial resolution of ~6.25 μm [27,34]. Using FTIR-I, the content and distribution of collagen and proteoglycans were mapped in the decalcified samples, while mineral content and distribution were determined across each insertion site in the PMMA-embedded samples. For each sample, three regions of interest (~400 x 1250 μm/region) were randomly selected for imaging and analysis, with each region spanning across the entire interface progressing from ligament to fibrocartilage and finally into bone, constituting about 10,000 points of spectral data acquisition per region analyzed or a total of 30,000 spectra collected per sample. Ligament, fibrocartilage, and bone regions were discerned based on tissue morphology evaluated using light micrographs (Figure 1-A) combined with histology.

### Spectral Analysis – Matrix Distribution and Collagen Alignment

The FTIR spectra were analyzed and spectroscopic images generated using ISYS 3.1.1 chemical imaging software (Spectral Dimensions Inc., Olney, Maryland, USA) and MATLAB 7.10 R2010b (The MathWorks Inc., Natick, Massachusetts, USA). Prior to analysis, spectra were corrected by baseline subtraction using the ISYS software. Collagen content was estimated by integrating the peak area under the Amide I band (1720-1590 cm^-1^), and glycosaminoglycan (GAG) content was estimated by integrating the area under a carbohydrate band associated with C-O-C and C–OH vibrations (1140-985 cm^-1^), according to previous studies that have correlated collagen and GAG content to these respective band areas (Figure 1-B) [21,22,28]. Although more recent studies have shown some improvement in specificity of proteoglycan (PG) assessment using a multivariate analysis methodology [37], the PG parameters used in the current study had previously been validated by correlation to both biochemical and histological data [21,38].

Additionally, collagen alignment was determined by scanning the demineralized samples with a gold-wire polarizer grid (PerkinElmer) inserted into the path of the IR light, with the polarizer aligned at 0° with respect to the interface between ligament and bone. As Amide I and amide II bond vibrations are approximately orthogonal [23], previous studies have shown that the ratio of their band areas is an indicator of collagen fibril orientation when spectra are collected under polarized light [24,28]. Therefore in this study, spectra obtained with the polarizer were integrated under the amide I (1720-1590 cm^-1^) and amide II bands (1590-1480 cm^-1^), and numerical indices for collagen orientation were obtained by calculating the amide I: amide II band area ratio. Collagen orientation was categorized according to the parameters previously reported for cartilage analysis and a 0^°^ polarizer setting, whereby amide I: amide II ratio values ≥2.7 and ≤1.7 indicate fibrils parallel and perpendicular to the bone-fibrocartilage interface, respectively, while a ratio ranging from 1.7-2.7 indicates mixed or random fibril orientation [24].

### Spectral Analysis – Mineral Distribution

The relative mineral-to-matrix ratio was calculated by integrating the area under the ν1, ν3 phosphate band contour (1200-900 cm^-1^) and dividing by the amide I band area (Figure 1-B). Prior to analysis of mineral distribution in transverse sections, the collected spectra were corrected for contributions of the PMMA embedding material [30]. Specifically, spectra of pure PMMA were acquired, baseline corrected, and normalized to the highest peak in the PMMA spectra (1728 cm^-1^). Sample spectra were likewise baseline corrected and normalized, and a pure PMMA spectrum was subtracted from the sample spectra to eliminate the PMMA background. This method of normalization and subtraction was implemented in order to compensate for different degrees of PMMA penetration into the multiple tissue types evaluated across the insertions.

### Line Profiles of Matrix and Mineral Distribution

To assess matrix and mineral distribution in the fibrocartilage interface, line profiles of collagen, proteoglycan and mineral extending across the fibrocartilage region progressing from ligament to bone, as identified based on tissue morphology assessed from corresponding light micrographs, were generated and values for 100 equally spaced points were interpolated using a MATLAB bicubic least squares method. This normalization method is advantageous since it allows for an expression of matrix content as a function of percent distance across the insertion, thereby accounting for any variation in fibrocartilage thickness between insertion samples. Line profiles were performed repeatedly across the interface spectroscopic maps on a pixel-by-pixel basis and then averaged, resulting in a single average line profile representing all the data collected for the fibrocartilage region. Regions exhibiting anomalies such as holes or folds in the sections were excluded. For region-dependent mineral content, tissue-specific mineral-to-matrix ratios were determined by averaging values for approximately 25 positions randomly selected within each interface region.

### Statistical Analysis

Results are presented in the form of mean ± standard deviation, with *n* equal to the number of specimens analyzed. Weighted averages, based on the dimension of each region of interest and corresponding number of individual line profiles, were calculated for each specimen, and the resulting profiles were subsequently averaged and standard deviation calculated for each group of specimens. Two-way analysis of variance (ANOVA) was performed to determine region- and insertion-dependent differences in matrix or mineral content. Tukey HSD post-hoc tests were performed for all pair-wise comparisons with significance declared for p<0.05. Statistical analysis was performed using the Minitab 16 statistical software package (Minitab, Inc., State College, Pennsylvania, USA).

## Results

### Effects of Sample Preparation on FTIR Spectra

**Figure 2:**
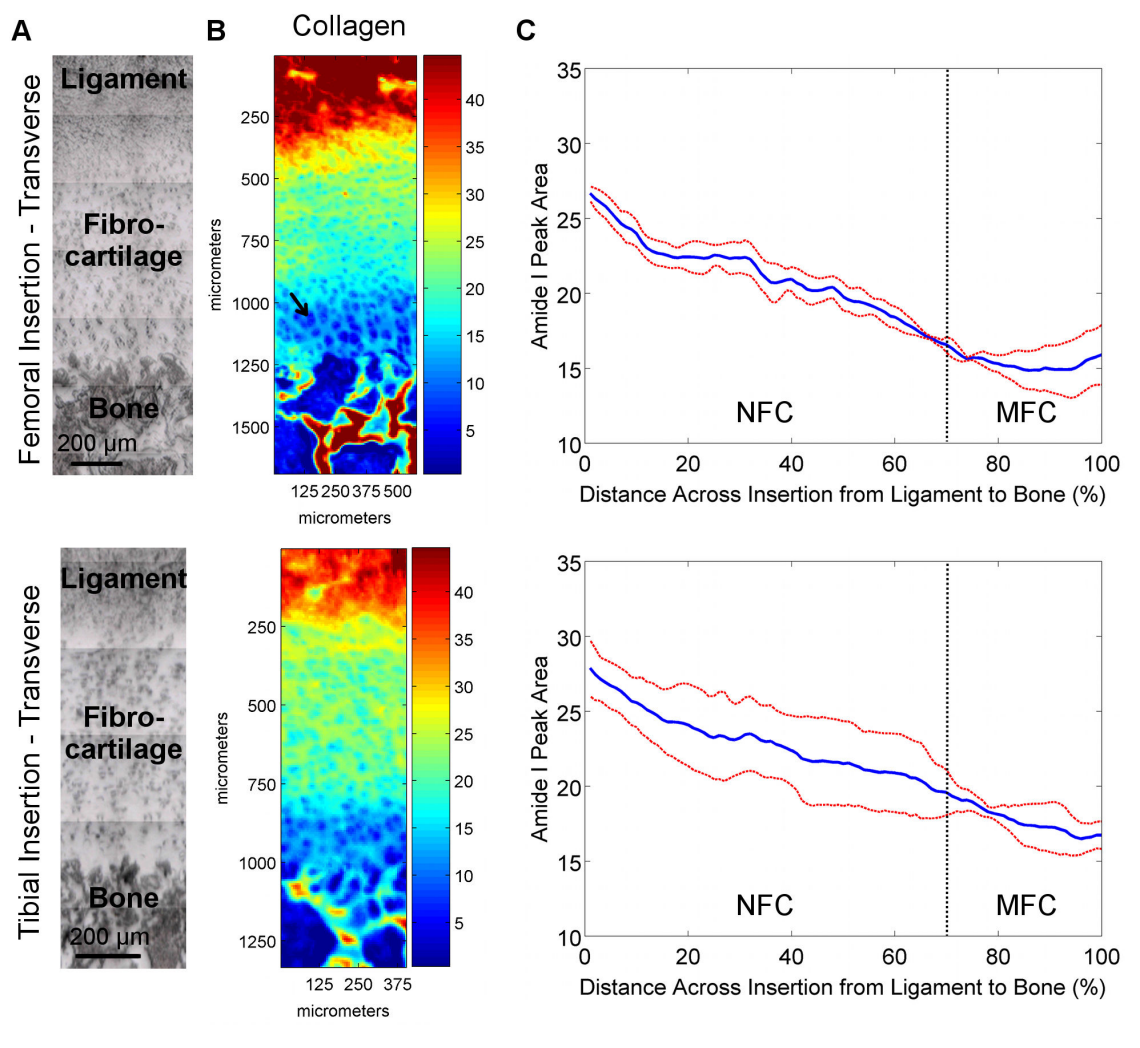
Collagen distribution in transverse sections of femoral and tibial ACL insertions. A) Light microscopy images showing ligament, fibrocartilage, and bone regions for which IR spectra were collected (bar = 200 μm). B) Corresponding spectroscopic maps of collagen content and distribution. Blue and red colors indicate low and high matrix content, respectively. High collagen content was found in the ligament and bone regions, with a gradual decrease in collagen observed within the fibrocartilage interface progressing from ligament to bone for both femoral and tibial insertions. C) Average collagen distribution within the insertion fibrocartilage, normalized for percent distance from ligament (0%) to bone (100%), revealing a gradient of collagen content across the fibrocartilage interface (*Blue* and *red* lines represent mean values and standard deviation, respectively; n=3; NFC = Non-Mineralized Fibrocartilage, MFC = Mineralized Fibrocartilage).

**Figure 3:**
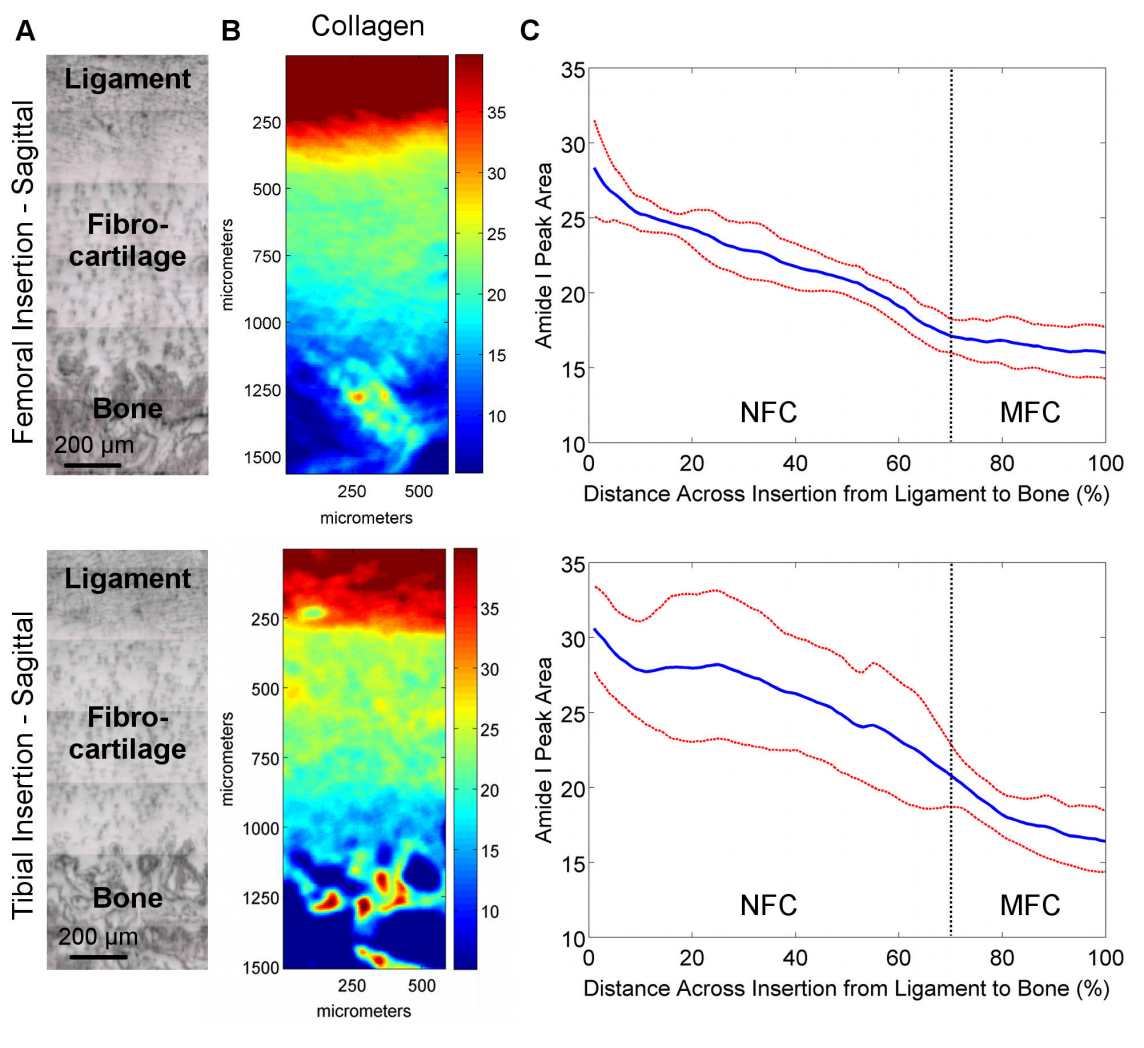
Collagen distribution in sagittal sections of femoral and tibial ACL insertions. A) Light microscopy images showing ligament, fibrocartilage, and bone regions for which IR spectra were collected (bar = 200 μm). B) Corresponding spectroscopic maps of collagen content and distribution. Blue and red colors indicate low and high matrix content, respectively. High collagen content was found in the ligament and bone regions, with a gradual decrease in collagen observed within the fibrocartilage interface progressing from ligament to bone for both femoral and tibial insertions. C) Average collagen distribution within the insertion fibrocartilage, normalized for percent distance from ligament (0%) to bone (100%), revealing a gradient of collagen content across the fibrocartilage interface (*Blue* and *red* lines represent mean values and standard deviation, respectively; n=3; NFC = Non-Mineralized Fibrocartilage, MFC = Mineralized Fibrocartilage).

**Figure 4:**
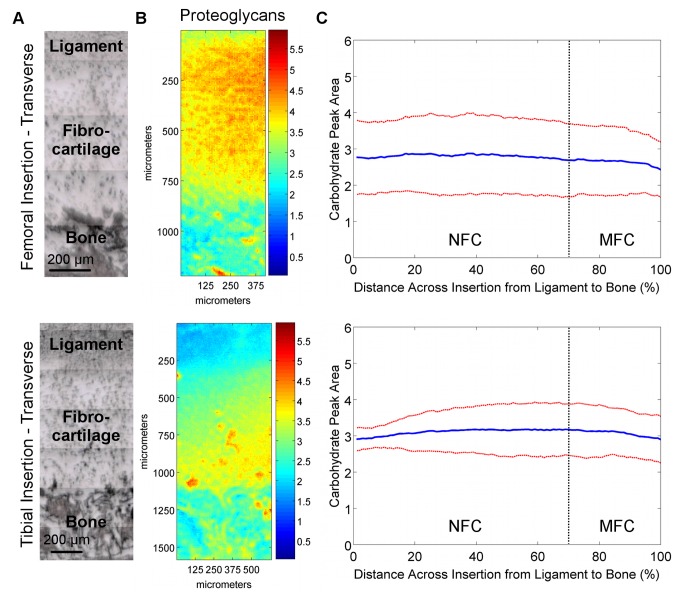
Proteoglycan distribution in transverse sections of femoral and tibial ACL insertions. A) Light microscopy images showing ligament, fibrocartilage, and bone regions (bar = 200 μm). B) Spectroscopic maps of proteoglycan distribution. Blue and red colors indicate low and high matrix content, respectively. Proteoglycan distribution was found to be highly variable, with regions of high and low PG content seen in both insertions. C) The high lateral variability in proteoglycan distribution is revealed by the large deviation (*red*) from the mean (*blue*) peak area values, and is more evident in the femoral insertion compared to the tibial insertion (n=3; NFC = Non-mineralized Fibrocartilage, MFC = Mineralized Fibrocartilage).

**Figure 5:**
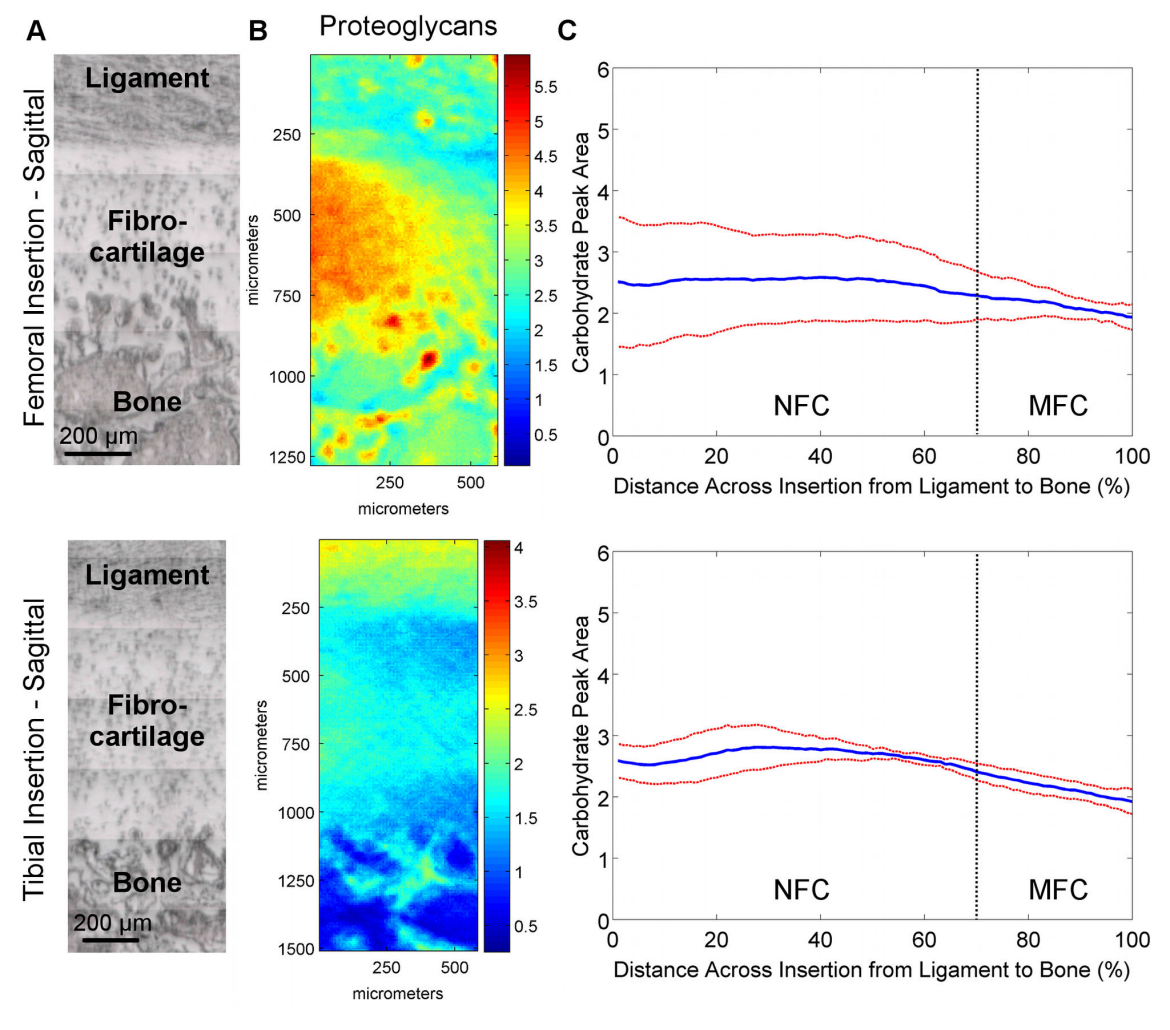
Proteoglycan distribution in sagittal sections of femoral and tibial ACL insertions. A) Light microscopy images showing ligament, fibrocartilage, and bone regions (bar = 200 μm). B) Spectroscopic maps of proteoglycan distribution. Blue and red colors indicate low and high matrix content, respectively. Proteoglycan distribution was found to be highly variable, with regions of high and low PG content seen in both insertions. C) The high lateral variability in proteoglycan distribution is revealed by the large deviation (*red*) from the mean (*blue*) peak area values, and is more evident in the femoral insertion compared to the tibial insertion (n=3; NFC = Non-mineralized Fibrocartilage, MFC = Mineralized Fibrocartilage).

Distinct spectra were obtained for ligament, fibrocartilage, and bone regions (Figure 1-C). Both Amide I (1720-1590 cm^-1^) and amide II (1590-1480 cm^-1^) peaks were evident in all three tissue regions. The carbohydrate band related to proteoglycans (1140-985 cm^-1^) was present in the fibrocartilage spectra, and an intense phosphate ν1, ν3 band (1200-900 cm^-1^) was evident in the bone spectra. No difference in collagen distribution was observed in the PMMA-embedded, non-decalcified sections when compared to the paraffin-embedded, decalcified samples, but the phosphate band was absent from the decalcified samples (data not shown). In addition, similar trends in collagen (Figure 2 and Figure 3) and proteoglycan (Figure 4 and Figure 5) distribution were found for both transverse and sagittal sections.

### Region-Dependent Matrix Distribution at the Insertion

#### Collagen Content and Distribution

Spectroscopic mapping of transverse (Figure 2-B) and sagittal (Figure 3-B) sections revealed that, compared to the fibrocartilage interface, the estimated collagen content from FTIR-I was higher in the ligament and bone regions. Collagen content also decreased progressing from non-mineralized to the mineralized fibrocartilage regions for both femoral and tibial insertions as indicated in the line profiles in Figure 2-C and Figure 3-C. A significant decrease in average collagen content was seen between 20% and 80% distance across the fibrocartilage interface for both types of insertions and for both transverse (Femoral: p=0.002, Tibial: p=0.006) and sagittal (Femoral: p=0.043, Tibial: p=0.011) sections. Line profile analysis (Figure 2-C and Figure 3-C) revealed a consistent decrease in relative collagen content across the fibrocartilage as evident by the small deviations, with overall about two-fold decrease in collagen content across the interface. Regression analysis of line profile data revealed that collagen decreased exponentially with a constant of (-5.8 ± 0.7) x 10^-3^, R^2^ = 0.96.

#### Collagen Orientation

**Figure 6:**
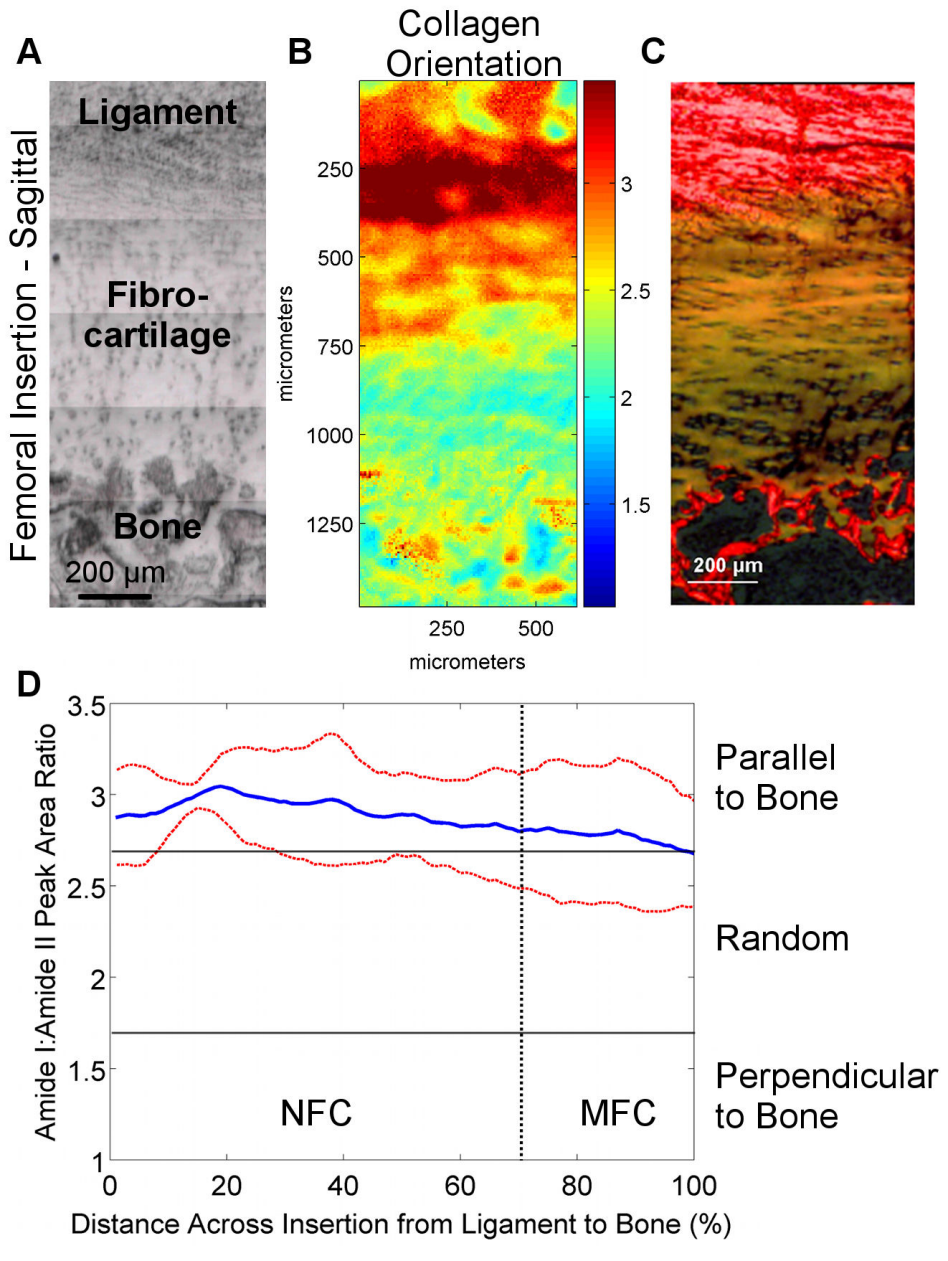
Collagen orientation in femoral insertions (sagittal samples). A) Light microscopy images showing the scan area (bar = 200 μm). B) Corresponding spectroscopic maps showing collagen orientation as determined by the amide I: amide II peak area ratio at 0° polarizer orientation. Values greater than 2.7 (*red*) and less than 1.7 (*blue*) indicate alignment parallel and perpendicular to the fibrocartilage-bone interface, respectively, whereas intermediate ratios (*light blue* to *orange*) indicate random orientation. C) Collagen orientation as visualized by polarized light microscopy of Picrosirius red histological stains. D) Average collagen orientation (0°) within the insertion fibrocartilage progressing from ligament to bone. Collagen fibers in the fibrocartilage region are initially parallel to the fibrocartilage-bone interface but obtain a more random orientation toward mineralized fibrocartilage and bone (*Blue* and *red* lines represent mean values and standard deviation, respectively; n=3; NFC = Non-Mineralized Fibrocartilage, MFC = Mineralized Fibrocartilage).

**Figure 7:**
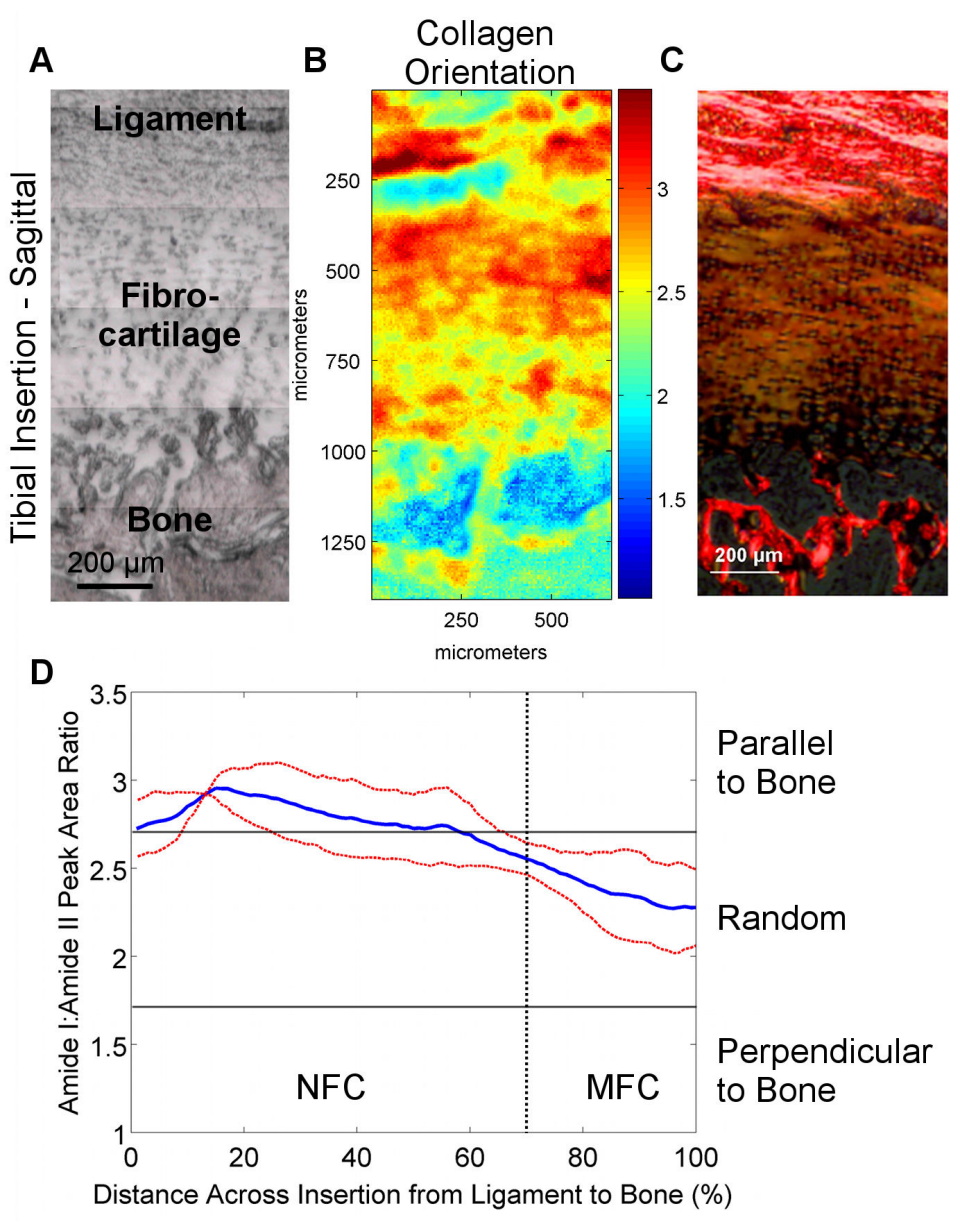
Collagen orientation in tibial insertions (sagittal samples). A) Light microscopy images showing the scan area (bar = 200 μm). B) Corresponding spectroscopic maps showing collagen orientation as determined by the amide I: amide II peak area ratio at 0° polarizer orientation. Values greater than 2.7 (*red*) and less than 1.7 (*blue*) indicate alignment parallel and perpendicular to the fibrocartilage-bone interface, respectively, whereas intermediate ratios (*light blue* to *orange*) indicate random orientation. C) Collagen orientation as visualized by polarized light microscopy of Picrosirius red histological stains. D) Average collagen orientation (0°) within the insertion fibrocartilage progressing from ligament to bone. Collagen fibers in the fibrocartilage region are initially parallel to the fibrocartilage-bone interface but obtain a more random orientation toward mineralized fibrocartilage and bone (*Blue* and *red* lines represent mean values and standard deviation, respectively; n=3; NFC = Non-Mineralized Fibrocartilage, MFC = Mineralized Fibrocartilage).

Sagittal sections were analyzed for collagen orientation since the insertion of collagen fibrils from the ACL into bone through the fibrocartilage interface can only be visualized in this orientation. Progressing across the fibrocartilage interface from ligament to bone, collagen fibrils were observed to initially be oriented parallel (Amide I:II ratio) to the fibrocartilage-bone interface. This orientation changed continuously, with a more mixed or random collagen fibril orientation (Amide I:II ratio) observed toward the center and MFC region of the fibrocartilage interface for both femoral (Figure 6) and tibial insertions (Figure 7).

#### Proteoglycan Content and Distribution

The proteoglycan content and distribution at the femoral and tibial insertions were highly variable. Specifically, proteoglycan content varied laterally throughout the fibrocartilage regions spectroscopically in both transverse (Figure 4-B) and sagittal (Figure 5-B) planes. Line profiles (Figure 4-C and Figure 5-C) revealed that on average, proteoglycan content was relatively constant across the fibrocartilage from ligament to bone, with slightly higher levels of proteoglycans seen in the center of the fibrocartilage compared to the interfaces with ligament and bone. The high lateral variability was evident by the large deviations, particularly in the femoral insertions.

### Region-Dependent Mineral Distribution

**Figure 8:**
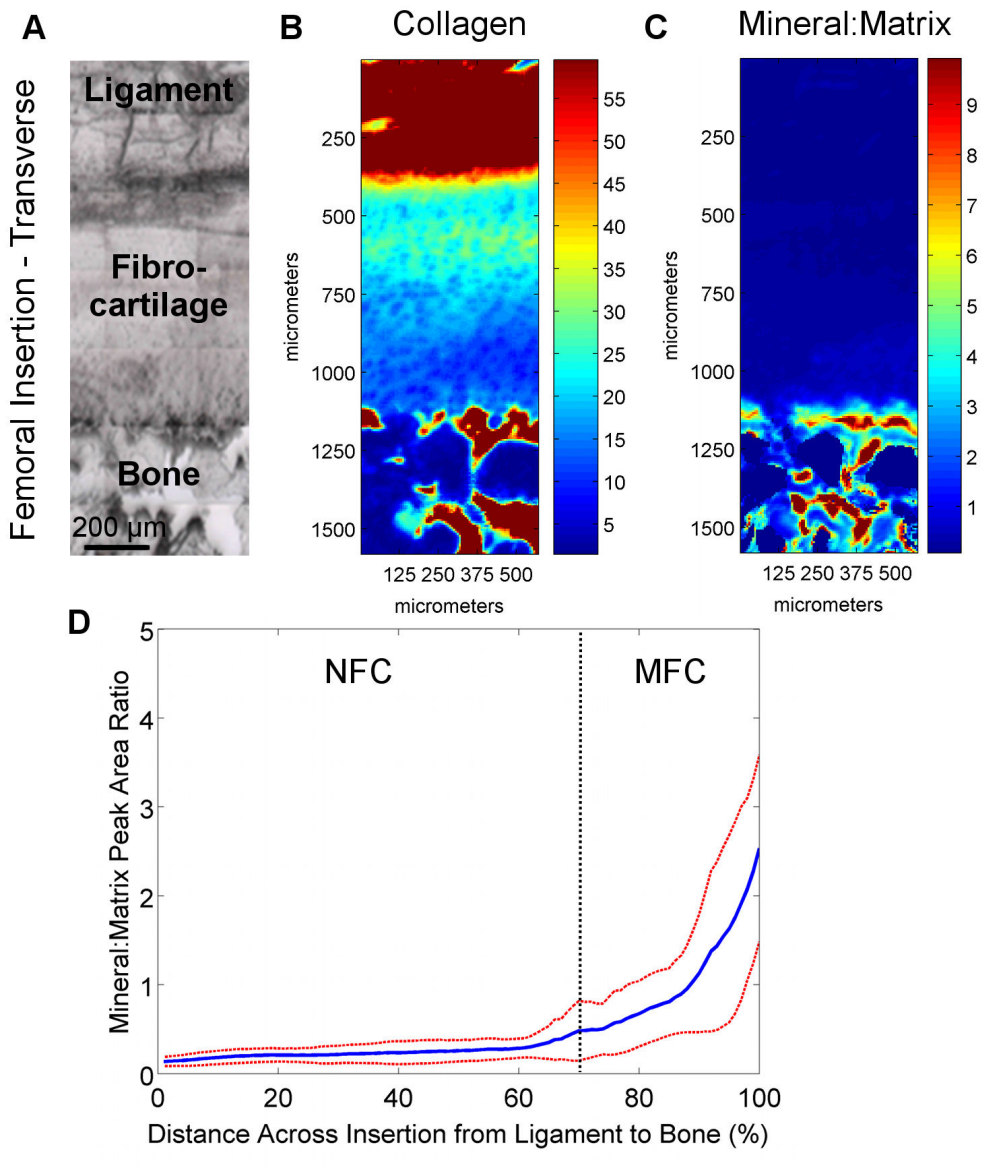
Collagen and mineral distribution across femoral insertions (transverse PMMA-embedded samples). A) Light microscopy images showing the scan area (bar = 200 μm). B) Confirming the analysis of the paraffin-embedded sections, high collagen content was again found in the ligament and bone regions, with a consistent decrease in collagen within the fibrocartilage interface progressing from ligament to bone. C) The mineral:matrix ratio was found to be high in the bone and mineralized fibrocartilage, with D) an abrupt change in mineral content between the non-mineralized fibrocartilage and mineralized fibrocartilage regions. *Blue* and *red* colors in the spectroscopic maps indicate low and high content, respectively (*Blue* and *red* lines represent mean values and standard deviation, respectively; n=3; NFC = Non-Mineralized Fibrocartilage, MFC = Mineralized Fibrocartilage).

**Figure 9:**
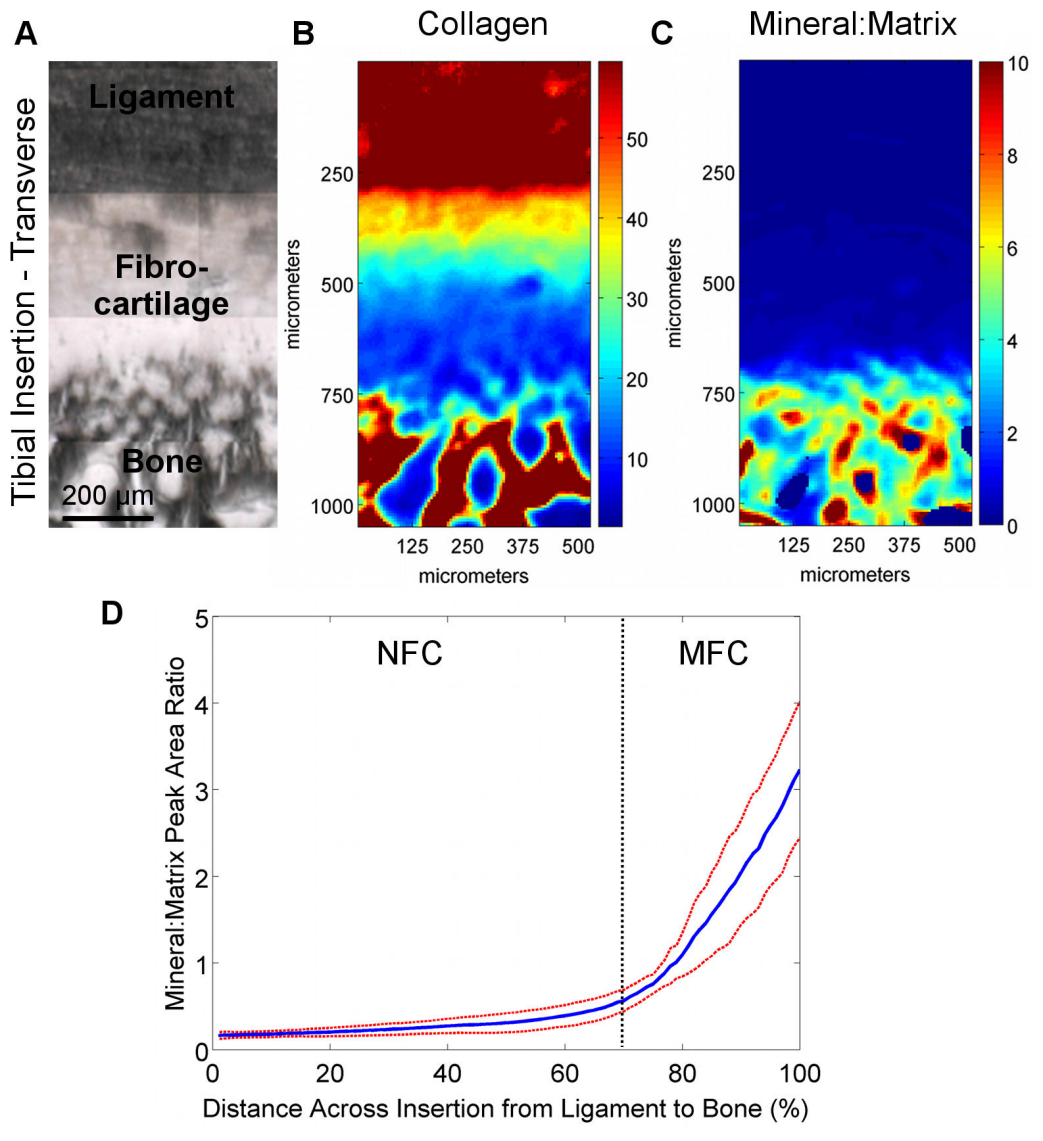
Collagen and mineral distribution across tibial insertions (transverse PMMA-embedded samples). A) Light microscopy images showing the scan area (bar = 200 μm). B) Confirming the analysis of the paraffin-embedded sections, high collagen content was again found in the ligament and bone regions, with a consistent decrease in collagen within the fibrocartilage interface progressing from ligament to bone. C) The mineral:matrix ratio was found to be high in the bone and mineralized fibrocartilage, with D) an abrupt change in mineral content between the non-mineralized fibrocartilage and mineralized fibrocartilage regions. *Blue* and *red* colors in the spectroscopic maps indicate low and high content, respectively (*Blue* and *red* lines represent mean values and standard deviation, respectively; n=3; NFC = Non-Mineralized Fibrocartilage, MFC = Mineralized Fibrocartilage).

**Figure 10:**
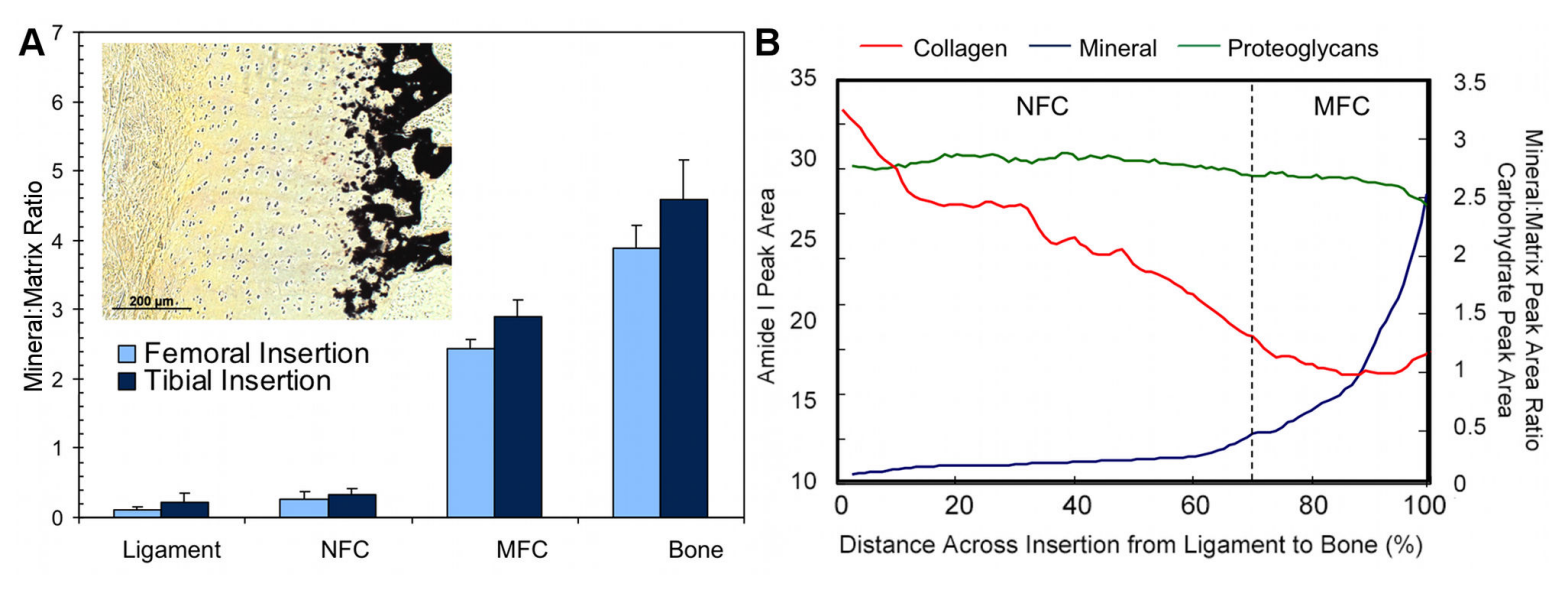
Region-dependent mineral content and overall matrix distribution across the ACL-bone interface. A) Mineral:matrix ratio was significantly higher in the MFC and bone regions compared to ligament and NFC for both femoral and tibial insertions (p<0.001); however, mineral content in MFC was significantly lower than bone (p<0.001). Average relative mineral content was higher in the tibial MFC (p=0.572) and bone (p=0.175) compared to the femoral insertion, although differences were not statistically significant. *Insert*: Mineral distribution across the ACL-bone insertion as shown by von Kossa histological staining confirms spectroscopic analysis (10x, bar = 200 μm). B) Collagen content (*red*) decreases across the fibrocartilage interface from ligament to bone, while mineral content (*blue*) increases and proteoglycans (*green*) remain relatively constant (Average data from transverse sections of femoral insertion; NFC = Non-Mineralized Fibrocartilage, MFC = Mineralized Fibrocartilage).

Region-dependent changes in the mineral-to-matrix ratio were detected at the interface. As expected, higher relative mineral-to-matrix ratios were found in the bone and mineralized fibrocartilage regions when compared to both the ligament and non-mineralized fibrocartilage regions (Figure 8 and Figure 9). An abrupt increase in mineral content was observed between the non-mineralized and mineralized fibrocartilage regions, as seen in the line profiles (Figure 8-D and Figure 9-D), as opposed to a gradual gradient in mineral content extending from ligament to bone. Based on average mineral distribution, obtained through line profile analysis, the mineralized fibrocartilage region accounted for approximately 30% of the fibrocartilage interface. Within this calcified region, regression analysis revealed that the mineral:matrix ratio increased exponentially (R^2^ = 0.98) with an exponential constant of (5.8 ± 0.3) x 10^-2^. In addition, the mineral-to-matrix ratio was significantly higher in the mineralized fibrocartilage and bone regions compared to the ligament and non-mineralized fibrocartilage regions (p<0.001, Figure 10-A).

### Insertion Site-Dependent Differences in Matrix and Mineral Distribution

Relative collagen content decreased across the femoral and tibial insertion fibrocartilage from ligament to bone, although the consistency, based on standard deviation, was found to be greater for the femoral insertion compared to the tibial (Figure 2-C and Figure 3-C). The location of the insertion (i.e. femoral, tibial) was found to have a significant effect on collagen content for transverse cuts (p=0.033); however, comparisons at either 20% or 80% distance across the fibrocartilage transition were not significant (20%: p=0.556; 80%: p=0.179). Relative collagen content was not significantly different between femoral and tibial insertion fibrocartilage for sagittal cuts (p=0.150). Regression analysis of line profile data of the transverse sample revealed that collagen decreased exponentially with constants of -6.0 x 10^-3^, R^2^ = 0.96 for the femoral and -4.9 x 10^-3^, R^2^ = 0.98 for across the tibial fibrocartilage regions. A similar trend was observed in the sagittal samples, with a constant of -5.9 x 10^-3^, R^2^ = 0.98 and -6.5 x 10^-3^, R^2^ = 0.93 for femoral and tibial insertions, respectively. In general, little difference in proteoglycan content and distribution was observed between femoral and tibial insertions, although variability was greater within the femoral insertion, with more consistent proteoglycan distribution observed at the tibial insertion site (Figure 4 and Figure 5).

Mineral distribution was similar between the femoral and tibial insertion sites, with a rapid increase in relative mineral content at the transition between non-mineralized and mineralized fibrocartilage (Figure 8 and Figure 9). Regression analysis of line profile data revealed that mineral:matrix ratio increased exponentially within the mineralized fibrocartilage region with constants of 5.5 x 10^-2^, R^2^ = 0.97 for the femoral and 6.0 x 10^-2^, R^2^ = 0.99 for the tibial insertion. In contrast, there was a trend toward higher average relative mineral content in both the mineralized fibrocartilage and bone regions of the tibial insertion site, although these differences were not statistically different (MFC: p=0.572; Bone: p=0.175; Figure 10-A).

## Discussion

Soft tissue to bone integration is crucial to the functionality of the musculoskeletal system, which is highly dependent on the transition of forces between tissues with distinct mechanical properties and functions. The objective of this study was to characterize the content, distribution, and organization of key matrix components across the multiple-region insertion. The results of this study represent the first quantitative mapping of matrix composition and distribution at the ACL-to-bone interface. Utilizing Fourier transform infrared imaging (FTIR-I), both region- and insertion-dependent variations in collagen, proteoglycan, and mineral content, as well as collagen orientation, were detected and quantified across the multi-tissue regions of the interface. Characterizing these compositional changes, as summarized in Figure 10-B, is crucial to elucidating the role of the multi-tissue interface in facilitating the transfer of load between soft and hard tissues.

Systematic analysis of femoral and tibial ACL insertions revealed region-dependent changes in collagen distribution, which is in agreement with published histological characterizations [3,4,6–8,10]. As expected, collagen content was the highest in the ligament and bone regions, and was the lowest in the fibrocartilage interface. Particularly interesting was the apparent decrease in collagen content within the fibrocartilage region progressing from ligament to bone. This decrease was found to be highly consistent among all of the samples analyzed. This decrease in matrix content is likely balanced by increases in hydration or overall cellularity and cell surface area across the interface [10]. Moreover, collagen alignment was found to vary across the fibrocartilage interface, with collagen fibers initially parallel to the fibrocartilage-to-bone interface and progressively more randomly oriented across the interface toward bone. A reduction in collagen fibril alignment at the soft tissue-to-bone interface have also been reported for the rotator cuff tendon-to-bone insertion in the rat shoulder [9], likely related to a change in loading profile from primarily tensile in the ligament or tendon, to a mixture of tension and compression at the fibrocartilage interface [18].

Region-dependent differences in proteoglycan content were also detected at the ACL-to-bone insertions. In particular, changes in proteoglycan content were evident laterally throughout the insertion fibrocartilage, although on average, little difference was seen across the fibrocartilage region from ligament to bone. The absence of large differences in proteoglycan content among the tissue regions or variations within the fibrocartilage itself may be related to the neonate state of the tissue. In addition, the type of proteoglycan and glycosaminoglycan chain length may vary between different regions and tissues, possibly also accounting for changes in total GAG content found laterally in the insertions. For example, Vogel et al. have shown that the primary proteoglycan in the tensile-loaded region of the human tibialis posterior tendon is decorin, which is a small proteoglycan with only one GAG chain of varying length, whereas compressed fibrocartilaginous regions of the tendon contain small proteoglycans including decorin and biglycan, as well as large proteoglycans [39]. This distribution of proteoglycans likely reflects non-uniform compressive forces throughout the insertions.

An exponential gradient or relatively step-wise change in mineral content was observed between the non-mineralized and mineralized fibrocartilage regions. This finding was in contrast to the linear increase in mineral content reported for mature rat supraspinatus-bone interface, as determined using Raman spectroscopy [25,26]. Given that both the ACL and supraspinatus exhibit direct insertions into bone, the differences in mineral profiles likely arise due to the inherent differences in the biomechanics of the shoulder and knee, along with other factors such as specimen origin or age. The significant increase in compressive modulus from the non-mineralized to the mineralized fibrocartilage region [11] may be attributed to the exponential increase in mineral content observed here, with minimal contributions from other matrix components such as collagen or proteoglycan. It is likely that the decrease in collagen content across the fibrocartilage transition may help to mitigate interfacial stress concentrations that may be caused by the rapid increase in mineral content, and the change in collagen organization is likely in response to complex loading profiles experienced at the insertion.

The controlled spatial distribution of collagen, proteoglycans, and mineral content across the ACL-bone interface (Figure 10-B) can result in anisotropic mechanical properties that may facilitate the transmission of load from ligament to bone. It has been suggested that the function of the fibrocartilage interface at direct insertions is to mediate load transfer through the translation of tensile forces within the ligament into compression and shear [3,5]. The existence of compression at the ligament-bone interface has been shown through finite element modeling of the medial collateral ligament femoral insertion by Matyas et al. [12] as well as experimentally at the neonatal bovine ACL insertions using ultrasound elastography [18]. Transmission of loads through compression and shear would suggest the presence of proteoglycans at the insertions as this matrix component is often found in tissues subjected to compression. Proteoglycans were found to be present in the fibrocartilage interface, roughly evenly distributed across the fibrocartilage region from ligament to bone, although the content varied laterally within the insertion. The high lateral variability may reflect non-uniformity in compressive loading within individual insertion sites in the relatively young animals used in this study.

Insertion site-dependent differences were also observed here. While lateral variations in proteoglycan distribution were observed in both femoral and tibial insertions, the differences were more pronounced in the femoral insertion. Conversely, collagen distribution was more consistent in the femoral fibrocartilage compared to the tibial insertion. These disparities may reflect differences in the mechanical loading profile between insertions as well as throughout each insertion site. In addition, although not statistically significant, average relative mineral content was found to be higher in the tibial mineralized fibrocartilage and bone regions when compared to the femoral insertion, which may contribute to the higher apparent Young’s modulus of the tibial insertion reported by Moffat et al. [11].

This study examined the ligament-to-bone interface in an immature model due to the need for an understanding of this stage of enthesis formation as interface regeneration will likely require the initial formation of a similar immature enthesis prior to maturation. In this study, collagen fiber orientation in neonatal insertion fibrocartilage was found to vary minimally within the insertion, and did not yet exhibit the orientation reported for the mature enthesis [10]. Collagen fibers were found to be oriented parallel to the fibrocartilage-bone interface initially, but then gradually became more randomly oriented as they progressed toward bone. This decrease in collagen orientation progressing toward bone may also in part be due to the presence of type X collagen, which exhibits a hexagonal conformation [40] and therefore cannot become aligned in the same manner as types I and II collagen. It is fully anticipated that the insertion site remodels with age, particularly the fibrocartilage interface, potentially in response to mechanical loading.

Similar to collagen distribution and orientation, it is likely that proteoglycan content will change as the interface remodels with age. Wang et al. observed markedly low staining intensity for proteoglycans in the ACL-to-bone insertions of mature animals compared to neonatal, especially as the interface progressed from a hyaline-like cartilage to fibrocartilage [10]. Region-dependent inhomogeneity in GAG content has also been shown in mature articular cartilage [27,28] and intervertebral disc [41]. In addition, histological analysis of the medial meniscal horn attachments, which likewise exhibit fibrocartilaginous insertions into bone, have revealed differences in glycosaminoglycan content between the anterior and posterior insertions that may reflect inherent disparities in the mechanical loading profile and compressive mechanical properties of each insertion [42]. These reports of differences in both collagen and proteoglycan distribution between immature and mature tissues, and between different soft tissue-to-bone insertions, collectively suggest that insertion fibrocartilage remodels with increasing age. Quantitative FTIR-I analysis of these changes in the ACL-bone interface will be performed in future studies.

While this is the first study to apply FTIR-I for interface characterization, it is noted that collagen content across the fibrocartilage insertion was estimated based solely on the amide I band area, under the assumption that variations in section thicknesses are minimal. This assumption is likely valid given the large number of data points collected and consistency among the specimens. It is emphasized that the analysis performed in this study is intended to provide an estimate of the relative amounts of matrix and mineral content rather than absolute amounts. While the proteoglycan and collagen spectra overlap within the fingerprint region (1800-800 cm^-1^) [37], it has been previously shown that the amide I and carbohydrate peaks are linearly proportional and that proteoglycans contribute only weakly to the amide I band [28]. In addition, it is recognized that characterizing the bovine ACL insertion is not fully representative of the human interface. It is emphasized here that of interest in this study are not the specific measured values, but rather the trend or observed gradients in matrix content across compositionally distinct tissue regions. The advantages of the bovine model are that, in addition to relevant large animal model comparisons to published FTIR-I studies [27,28], bovine tissues are more readily available for study compared to those from humans, and samples can be obtained from young and healthy animals.

As the soft tissue-bone interface is crucial for musculoskeletal functionality, the findings of this study are particularly relevant for current efforts to achieve biological graft fixation by engineering a functional interface between ACL grafts and bone [43–46]. From a scaffold design perspective, the exponential increase in mineral content can be represented as a step-wise increase in scaffold mineral content in a stratified design with contiguous mineral-free and mineral-containing regions. In addition, in order to evaluate the effectiveness of scaffolds and interface regeneration strategies, it is crucial to evaluate the early healing response and interface maturation, as represented in the young animal tissue evaluated in this study. Collectively, the results of this study enhance the current understanding of the complexity of soft tissue-to-bone insertions and provide critical benchmarks for interface regeneration, as well as for ultimately integrative and functional soft tissue repair.

## Conclusions

This study focused on quantitative mapping of changes in matrix and mineral distribution across the ACL-to-bone insertion sites using the FTIR-I. Both region- and insertion site-dependent differences in collagen, proteoglycan, and mineral distributions were observed. These controlled spatial changes in matrix content and distribution likely contribute to the reported increases in mechanical properties across the insertion site, and facilitate the transition of complex loads from ligament to bone.
